# Case Report: A developmental and epileptic encephalopathy 45 due to *de novo* variant of *GABRB1*

**DOI:** 10.3389/fped.2024.1346987

**Published:** 2024-04-03

**Authors:** Lu Wang, Haiquan Xu, Jianbo Shu, Dandan Yan, Dong Li, Chunquan Cai

**Affiliations:** ^1^ Tianjin Pediatric Research Institute, Tianjin Children’s Hospital (Tianjin University Children’s Hospital), Tianjin, China; ^2^ Tianjin Key Laboratory of Birth Defects for Prevention and Treatment, Tianjin, China; ^3^ Department of Neurology, Tianjin Children’s Hospital (Tianjin University Children’s Hospital), Tianjin, China

**Keywords:** children, *de novo* variant, epilepsy, *GABRB1*, case report

## Abstract

**Background:**

The gamma-aminobutyric acid (GABA) variant causes developmental and epileptic encephalopathy 45 (DEE45), an autosomal dominant disorder that results in oculocortical visual impairment, reduced muscle tone, psychomotor retardation, and epilepsy. Analysis of the clinical features and genetics of DEE45 may be helpful in complementing genotype-phenotype studies.

**Case presentation:**

We collected peripheral blood samples from the affected children and parents and extracted genomic DNA. Whole exome sequencing (WES) was utilized to identify the underlying disease-causing variant. WES showed that the prior carried a heterozygous variant c.686C > T p.(Ala229Val) in exon 7 of the *GABRB1* (NM_000812.4), and no variant was detected in either parental sample. The child has DEE45.

**Conclusion:**

The variant c.686C > T of the *GABRB1* is a possible cause of DEE45. Gene variant analysis of the relevant family lines using WES provides effective genetic counseling for developing and regressing such patients in the clinic. However, further studies are needed to verify the pathogenic mechanism.

## Introduction

Epilepsy is a common neurological disease in children. With the application of genetic testing in clinical practice, an increasing number of children are being diagnosed with hereditary epilepsy. In hereditary epilepsy, most genetic variations are found in ion channels such as calcium, potassium, sodium, ligand-gated γ-aminobutyric acid type A receptor, and chloride channels. These ion channel variations can cause epilepsy through a series of complex mechanisms. These ion channel variations can alter the function of channel proteins through a series of complex mechanisms, leading to an abnormal increase in neuronal cell excitability, ultimately leading to seizures (1). The 2017 International League Against Epilepsy (ILAE) classification added the concept of developmental and epileptic encephalopathy (DEE), a clinically and genetically heterogeneous group of age-dependent neurological disorders characterized by intractable seizures in infancy or early childhood, with frequent epileptic activity and an underlying genetic etiology associated with developmental disorders or regression (2).

The rapid development of epilepsy genetics research in recent years has led to the successive discovery of a variety of epilepsy-associated gamma-aminobutyric acid (γ-GABA) receptor genes and further studies on their functions, which have led to an in-depth understanding of the pathogenetic link between increased neuronal excitability and epilepsy, thus facilitating progress in the diagnosis and treatment of epilepsy (3). In this study, we reported a case of developmental and epileptic encephalopathy 45 (DEE45) due to harboring a heterozygous variant in the *GABRB1*, which was a *de novo* variant. We investigated the *GABRB1* variant by WES, analyzed the clinical and genetic features of this case, and extended the genotype-phenotype study to provide effective genetic counseling for the development and regression of this type of patient in the clinic and to provide a better reference plan for treatment.

## Materials and methods

### Study proband

The child, a 4-month-old boy, was admitted to the hospital with the main reason of “intermittent convulsions for three months”. Examination results: length-66 cm, weight-7.1 kg, head circumference-40 cm. Fontanel is flat and soft, approximately 0.5 cm × 0.5 cm. Neurological examination of the neck is normal and reflexes are present. Childbirth history: The mother was in good health during pregnancy, and the child was born in the first trimester and delivered by cesarean section at full term (voluntary). Three months before admission (1 month of age), the child developed convulsions, which appeared when he first fell asleep and manifested as loss of consciousness, squinting eyes, and flexion and tonicity of the extremities. There were two convulsions, each lasting for a few seconds and then relieved; the interval between the two convulsions was about four days, and the child was given no special treatment. Eleven days before admission, the child had a second convulsion, which occurred about 30 min after falling asleep and when he first woke up. This time, there were 2 types of convulsions. The first manifestation was the same as before, with both eyes squinting to the left and right, the right eye being the predominant one, each lasting 5–10 s and then relieving on its own, with more than 10 episodes per day, the shortest interval being 1 h and the longest interval being 4 h, with a total of more than 100 episodes; the second manifestation was loss of consciousness, rhythmic eyebrow raising, frequent blinking, and corner of the mouth shaking, with no abnormalities in the limbs, each lasting about 1 min and relieving, with a total of 2 episodes. **Previous history**: no previous history of head trauma; **Family history**: Healthy parents; The two uncles of the father of the child had mental retardation, which was mainly reflected in speech disorder, social disorder, poor mathematical calculation ability, daily autonomous activities, and no convulsion. The uncle of the child has a history of intermittent convulsions, onset in adulthood, manifested as systemic tetanic seizures, lasting about a few minutes, can be self-relieved, normal intelligence, normal imaging examination, not perfect genetic examination, no drug treatment.

### Diagnostic

In this case, the child had recurrent epileptic seizures from the age of 1 month after birth, and the seizure forms were mainly generalized tonic-clonic and focal seizures generalized to the whole body, with more than two seizure forms. Based on the results of the Video electroencephalography (EEG), the highly dysrhythmic waves during the seizures were detected, and the imaging showed callus thinning, which is consistent with the typical features of DEE. Furthermore, after analyzing the information from the WES test, the diagnosis of DEE45 was confirmed.

### Differential diagnosis

Epileptic encephalopathy needs to be differentiated from the following diseases:

(1) Central nervous system infection: acute onset, usually with a history of antecedent infection; neurological examination can reveal typical signs; pathogenic test can find the corresponding pathogenic microorganisms; anti-infective treatment is effective, and the diagnosis is most often clarified by lumbar puncture examination and pathogenic examination;
(2) Electrolyte disorders and hypoglycemia: most of them have an acute onset, and laboratory biochemical tests are helpful for diagnosis, but neurological conditions need to be excluded;
(3) Inherited metabolic diseases: acute or subacute onset of illness, mainly with a history of stunted growth or feeding difficulties; the corresponding genetic metabolic screening can help to clarify the diagnosis but needs to exclude parenchymal organ diseases;
(4) Intracranial space-occupying lesions: most of them have an acute onset, and the neurological examination may show obvious positive signs, and the diagnosis can be clarified by imaging examination.

### Therapeutic interventions

**Figure 1:**
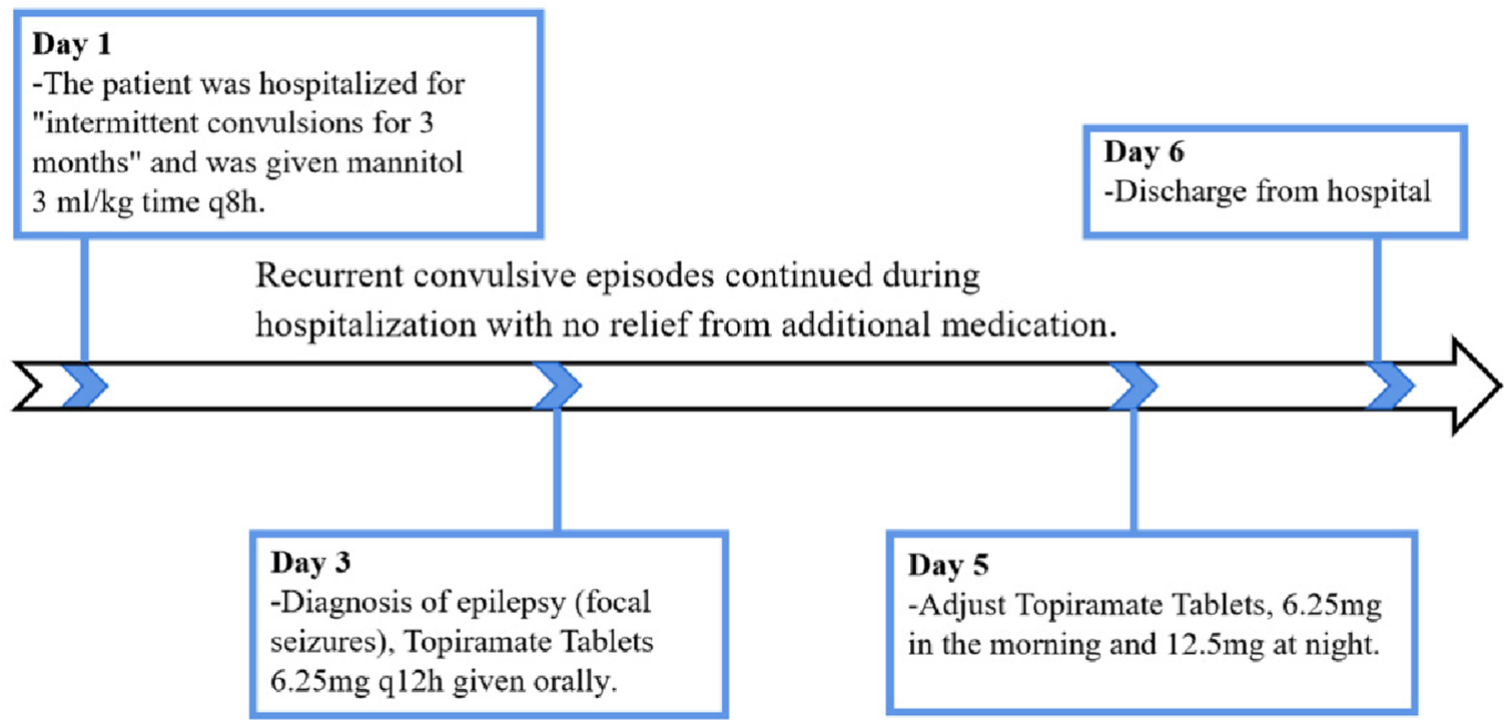
Timeline of the patient's clinical journal.

The child was treated with mannitol 3 ml/kg.time q8 h to reduce cerebral edema since the 1st day of hospitalization. On the 3rd day of hospitalization, a preliminary diagnosis of epilepsy (focal seizures) was made based on clinical manifestations, biochemistry, imaging, video EEG, etc., and topiramate [It can antagonize GLUR5 receptor and can also act as a the gamma-aminobutyric acid (GABA) receptor-mediated current-positive modifier] 6.25 mg q12 h was added to the oral treatment. There were still recurrent convulsive seizures, with the same manifestations as before. On the 5th day of hospitalization, topiramate was adjusted to 6.25 mg in the morning and 12.5 mg in the evening. There was no improvement in the symptoms after the addition of the drug, and the family requested to be discharged from the hospital on the 6th day of hospitalization (Figure 1).

### DNA extraction and WES

After the informed consent of the child's family, 2 ml of peripheral blood samples (ethical review approval number: 2022-LXKY-012) were collected from the child and his parents and stored at 4°C. The DNA was extracted using a Beijing Kangwei Century Biotechnology Co., Ltd. kit and held at −20°C. The samples were subjected to whole exome sequencing by the platform of Beijing Ajitaikang Biotechnology Co. Sequence comparison of the sequenced raw data was performed using BWA software with the hg19 human reference genome pair, and variant analysis was performed using GATK software, and annotation information from databases such as HGMD, dbSNP, OMIM, and Thousand Genomes was added using Annovar software.

## Results

### Routine examination results

**Figure 2:**
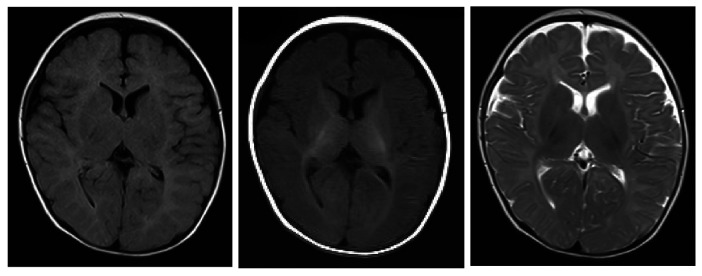
The extracerebral space was widened and the mucosa of the superior frontal and septal sinuses was thickened bilaterally.

**Figure 3:**
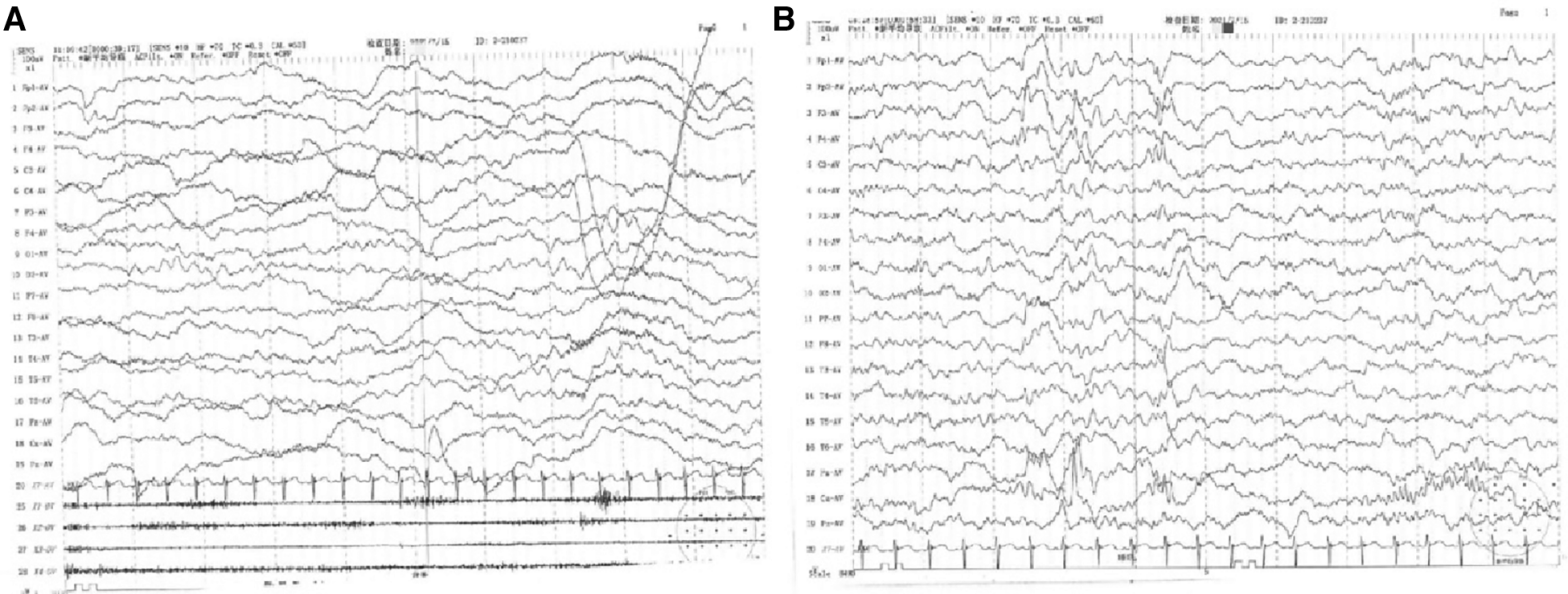
(**A**) 4–5 Hz low-moderate wave amplitude theta activity in the occipital region bilaterally in the awake and quiet closed-eye state, interspersed with a small amount of low amplitude fast waves, roughly symmetrical between left and right. (**B**) Abnormal electroencephalography (EEG) with bilateral frontal, left anterior temporal, parietal, and frontal midline area spike-wave and spike-slow wave emanations during sleep, with left-right asynchrony. Slightly more extensive low-amplitude fast waves in each conductor during the waking periods of sleep.

**Figure 4:**
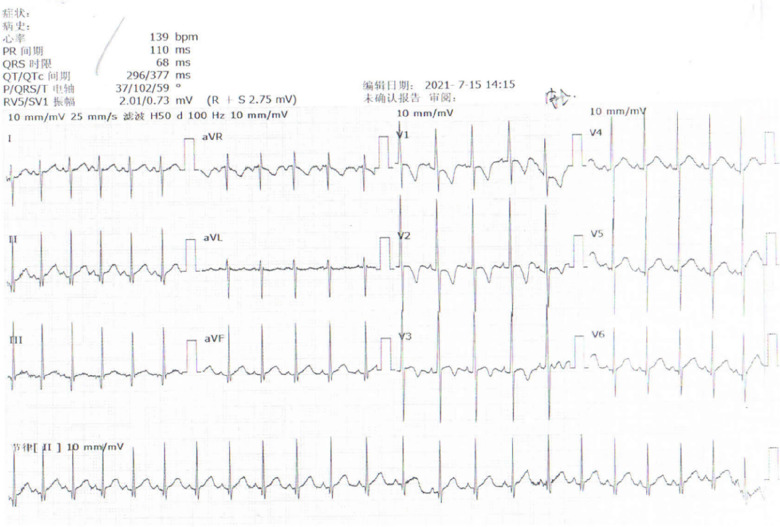
ECG shows sinus arrhythmia with non-specific ST elevation.

The child was a four-month-old boy who was admitted to the hospital for intermittent convulsions. During hospitalization, the child's head magnetic resonance imaging (MRI) showed no significant abnormalities (Figure 2); the brain waves were abnormal, with spikes and spikes and slow waves emanating from the bilateral frontal, left anterior temporal, parietal, and frontal midline regions during sleep, with left-right asynchrony (Figure 3); the Electrocardiograph (ECG) showed sinus arrhythmia with non-specific ST elevation (Figure 4); the urine gas phase mass spectrometry results showed no abnormalities, and the blood tandem mass spectrometry results showed increased C18:1 and decreased C8/C10. Combined with the urine screening results, the above changes were considered to be caused by abnormal liver function or nutritional medication. However, abnormal long-chain fatty acid metabolism and suspicion of CPT2 must be excluded.

### WES result

**Figure 5:**
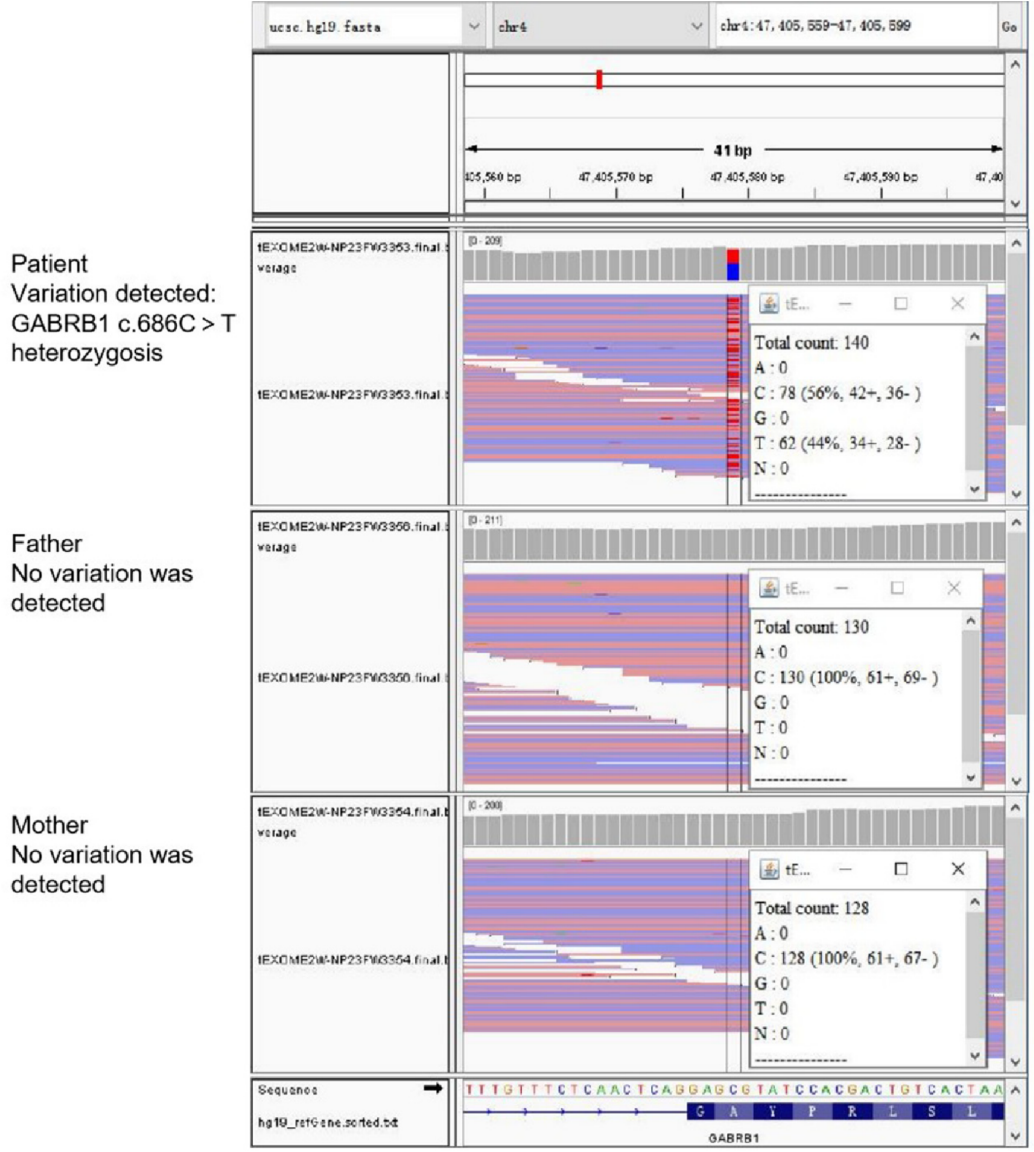
WES results. The prior carried a heterozygous variant and no variant was detected in either parental sample.

The pre-documented individuals carried the heterozygous variant c.686C > T p.(Ala229Val) in exon 7 of the *GABRB1*, and no variant was detected in either parental sample. No other pathogenic variants were detected by WES (Figure 5). Full-length sequencing of the mitochondrial ring DNA of the prior did not detect pathogenic variants associated with clinical cues.

### Pathogenicity analysis of *GABRB1* variants

This variant was not included in the professional version of the Human Gene Mutation Database (HGMD) and was searched as an unreported new variant; it was not detected in the parents and was presumed to be a *de novo* variant. According to the relevant guidelines of the American College of Medical Genetics and Genomics (ACMG), the variant c.686C > T of the *GABRB1* is consistent with the evidence of pathogenicity PS2: De novo variants in patients with no family history (verified by both parents); PM2: no variants found in normal control populations in the ESP database, “the 1,000 Genomes Project” database, and the EXAC database variants found. The variant was judged to be potentially pathogenic based on the above evidence when “1 strong (PS2) and 1–2 medium (PM2)” were met.

### Bioinformatics analysis

**Figure 6:**
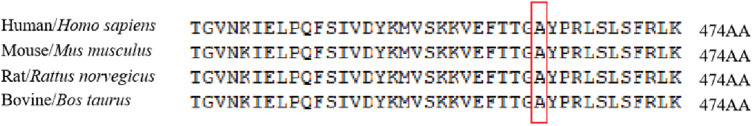
The homologous sequence of *GABRB1* was highly conserved at position 229 among different species.

**Figure 7:**
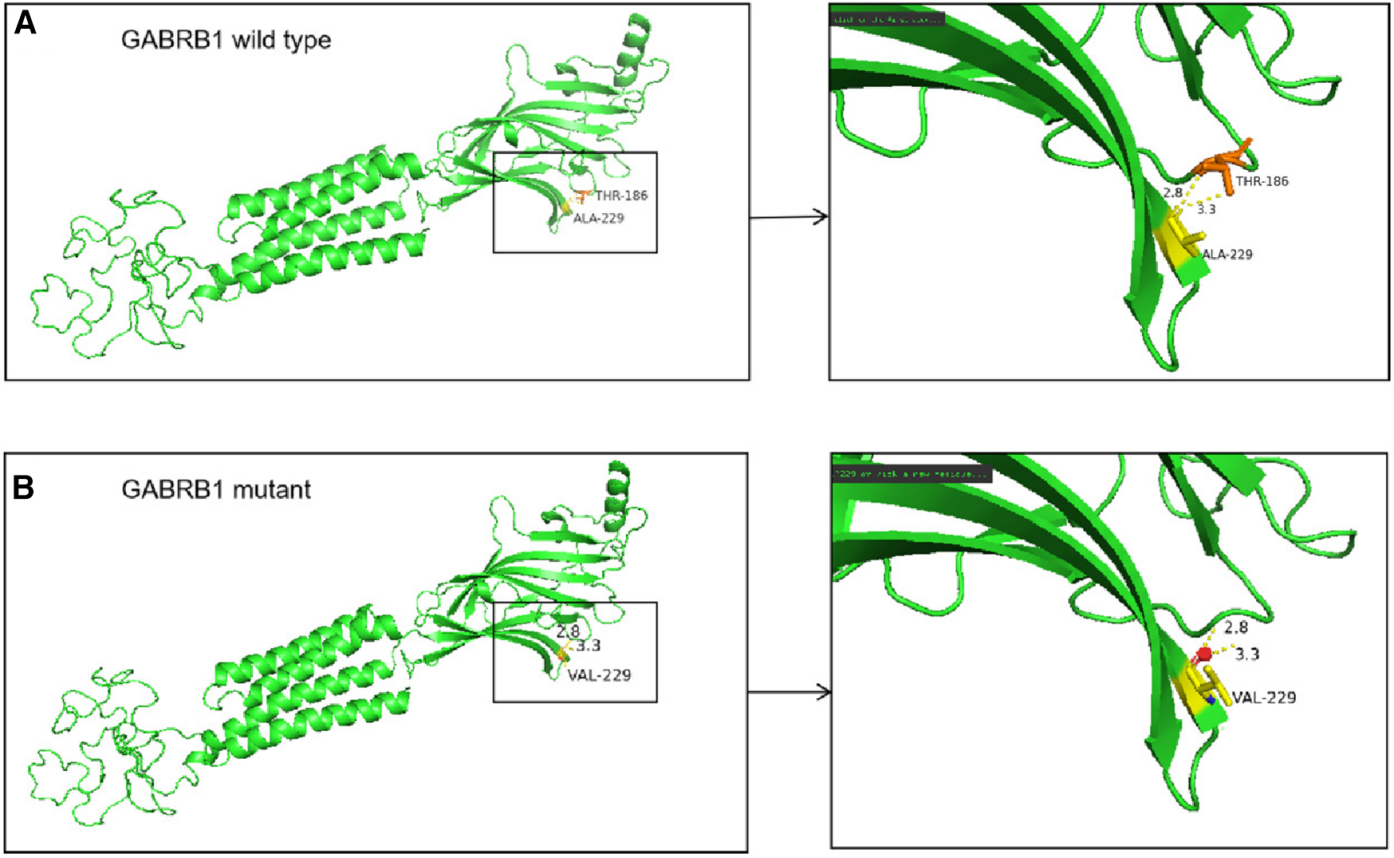
(**A**) In the wild-type protein, Ala229 is linked to Thr186 by two hydrogen bonds. (**B**) In the mutant protein, the alanine at position 229 is replaced by a valine, but no new secondary structure is formed.

Predictive software analysis showed that variant c.686C > T of the *GABRB1* was pathogenic (Table 1); amino acid sequence alignment showed that variant c.686C > T was highly conserved across the four species (Figure 6). 3D model predictions indicate that mutants do not form new secondary structures and do not introduce new hydrogen bonds (Figure 7).

**Table 1:** Bioinformatics software prediction of the deleteriousness of *GABRB1* variants found in this case study.

Genetic variant	Protein change	Type of variant	Results				
				Mutation taster	Polyphen-2	PROVEAN	SIFT
c.686C > T	p.(Ala229Val)	Missense	Score	0.999	0.944	−1.59	0.240
			Result	Disease causing	Possibly damaging	Neutral	Tolerated

### Follow up

After discharge, the child's family did not come to the hospital at the prescribed time to review the visit, the child's family could not be reached by telephone at a later stage, and follow-up information was lost. Unfortunately, long-term follow-up on disease prognosis is not available.

## Discussion and conclusions

With the application of WES technology, more and more genes are associated with DEE. 30% of DEE is caused by hereditary factors, with genes encoding neuronal ion channels or involved in synaptic transmission making up a significant portion of the population, such as *CACNA1E, SCN1A, KCNT1* et al. (4). Most children with epilepsy start within one year of age and initially have partial or complex partial seizures, while other forms of seizures include epileptic spasms, myoclonus, generalized tonic clonus, atonia, and atypical disorientation.

To date, 57 genes are associated with early infantile epileptic encephalopathy (EIEE) (http://www.omim.org/), with the most studied being the ion channel class of genes and the neurotransmitter receptor class of genes. GABA is the major inhibitory neurotransmitter in the mammalian central nervous system and is widely distributed throughout the nervous system in the cortex, thalamus, hippocampus, and amygdala (5). It has been clinically demonstrated that epilepsy and Posttraumatic stress sleep disorder are associated with reduced GABA-mediated inhibitory synaptic transmission, and that medications that enhance GABA-mediated inhibitory synaptic transmission in the CNS are effective not only in the treatment of anxiety and insomnia, but also in the treatment of epilepsy (6).

In 2013, cases of epilepsy associated with variants in the *GABRB1* were first reported. A variant of the GABA_A_ receptor β1 subunit was functionally described in 2016 in a case of epileptic encephalopathy (7), where the missense variant, an F246S variant and affecting chloride channel kinetics, occurred in the first helix of the transmembrane region of the β1 subunit in a child diagnosed with epilepsy from the age of 1 year, with infantile spasms from the age of 3 years and an overall developmental delay (7, 8). Lien (9) reported a second patient with epileptic encephalopathy and identified variants in the *GABRB1* as the cause of severe epilepsy. The child was diagnosed at three months of age with refractory epilepsy and severe developmental delay and carried a *GABRB1* variant (c.860C > T/p.Thr287Ile). This missense alteration occurs in the second helix of the transmembrane region of the β1-subunit, formally identifying variants in the *GABRB1* as the cause of severe epilepsy. Subsequently the relationship between *GABRB1* variants and epilepsy gradually attracted attention.

According to previous literature, 80.0% (40/50) of patients with DEE had seizures, and the median age of the first seizure was six months of age; 72.5% (29/40) of them had two or more seizure forms, 55.0% (22/40) had epileptic spasmodic seizures more often than not, focal motor seizures, generalized tonic seizures, and tonic seizures were 32.5% (13/40), 30.0% (12/40), 27.5% (11/40), and myoclonic seizures and focal impaired consciousness seizures were 25.0% and 20.0%, respectively (10). Variants in the *GABRB1* may result in epilepsy syndromes ranging from mild to severe, as do variants in the *GABRB3* (8, 11, 12). This child presented with recurrent seizures from 1 month of age, with more than two seizure forms, mainly generalized tonic-clonic and focal generalized generalized seizures. The video EEG captured highly dysrhythmic waves during the seizures, and the imaging showed thinning of the corpus callosum, which was typical of DEE. However, the age of onset of the disease was much younger than previously reported in the literature, and the early onset provided additional prerequisites for later developmental delay.

γ-GABA is the major inhibitory neurotransmitter in the mammalian central nervous system, which may affect the function of approximately half of all central neurons (13). GABA in the brain binds to receptors on the postsynaptic membrane, forming receptor complexes and changing the conformation, which activates ion channels, allowing selective passage of ions, causing hyperpolarization of neurons, inhibiting excessive firing of excitatory neurons, and ultimately acting as a barrier to neural signaling (5). Previous literature has not explored the changes in hormone levels in the case in question, and genetic metabolic blood screening and urine screening in the child in this case suggested increased C18:1 and decreased C8/C10. In conjunction with the urine screening results, the above changes are considered to be due to abnormal liver function or nutritional medication. However abnormal long-chain fatty acid metabolism and suspicion of CPT2 need to be ruled out. The liver function of this child was not abnormal, and no special nutritional drug treatment was given at the time of completing this examination. Based on the theory that hormones may affect the influence of GABA_B_ receptor expression, the relevant metabolomics examination should be improved to provide a more theoretical basis for the clarification of the influence of metabolic pathway abnormalities on the role of the GABA_B_ receptor. The metabolic abnormalities of the children also provide more adverse factors for their subsequent developmental delay and refractory epilepsy, suggesting that clinical attention should be paid to the monitoring of metabolic substances in these patients, which is beneficial to the grading of the disease and the assessment of the prognosis.

As of 2020, a total of nine variants in the *GABRB1* have occurred, including eight missense/nonsense variants and one small deletion. In this paper, we examined a child with epilepsy and his parents by WES. We identified the child as carrying a missense variant of the *GABRB1* c.686C > T p.(Ala229Val), which was not detected in either parent. Pathogenicity classification analysis: (1). This mutation has not been reported in the population database, which is a rare mutation and belongs to the evidence of moderate pathogenicity (PM2). (2). The mutation was not detected in the sequencing data of the parents, which was analyzed as a new mutation in the children, belonging to the evidence of strong pathogenicity (PS2). (3). Pathogenicity rating Combined with the above variation evidence and classification evidence, according to the ACMG guidelines, there is one medium pathogenicity evidence and one strong pathogenicity evidence: 1PS + 1PM, and this variation belongs to the possible pathogenic variation. Two uncles of the father of the child had intellectual disabilities, mainly manifested as speech disorders, social disorders, poor mathematical calculation ability, poor daily autonomous activities, and no convulsions. The uncle of the child has a history of intermittent convulsions, adult onset, manifested as systemic seizures, lasting a few minutes or so, can be self-alleviated, normal intelligence. The age and form of onset of the disease are different from that of the patient in this case, and the difference suggests that the genetic factors of the disease have a small familial relationship. Unfortunately, family members other than the parents were not sequenced for the whole exome, so it was impossible to determine whether there was a variant in the epilepsy-related gene. It is suggested that children with early seizures in the clinic should record family history details in detail, improve genetic examination, and further provide validation experiments if conditions permit, so as to provide a more effective evaluation means for determining neonatal variation and pathogenicity.

This variant is located in exon 7, using bioinformatics analysis prediction software suggests that the c.686C > T variant of the *GABRB1* is pathogenic. Amino acid sequence comparison results show that the c.686C > T variant is highly conserved among the four species, and has not been reported in the HGMD database as of 2020 and that this site is a new causative locus, but 3D modeling predictions show that the mutant does not form a new However, 3D model predictions show that the mutant does not form a new secondary structure and does not introduce new hydrogen bonds, suggesting that the variant does not cause spatial structural changes. During hospitalization, the child was treated with topiramate, which exerts its antiepileptic effect by increasing the frequency of GABA activation of the GABA_A_ receptor, enhancing the inward flow of chloride ions, and increasing the level of inhibitory central neurotransmitters. However, the child in this case had a poor response to medication and continued to have seizures and poor symptom control while on medication. Although the correlation between topiramate blood concentration and its clinical efficacy should be noted, it is still necessary to be alert to the possible factors affecting the expression of GABA_B_ receptors without significant changes in the spatial structure, such as the regulation of the metabolic pathway or the involvement of endocrine function axis and its mediation.

The limitation of this study is that the long-term follow-up results were not collected, which limited the integrity of the study, suggesting that the complete acquisition of clinical follow-up information is an important guarantee for the realization of the study cycle analysis, especially for the follow-up of special cases at the early stage of the disease, it is necessary to strengthen the adequate communication and emotional care for family genetic counseling, which may be a potential strategy to improve the follow-up rate of genetic diseases. The limited availability of biological samples in this study suggests that in the subsequent clinical work, attention should be paid to the collection and preservation of samples and the improvement of functional verification experiments, so as to provide strong evidence for resolving the uncertainty of genetic analysis.

In conclusion, we analyzed the clinical characteristics and genetics of this case, so as to expand the genotype-phenotype study, and provide genetic counseling suggestions for the development and regression of this type of patients in clinical practice. The exploration of this new mutation is expected to provide certain references for the treatment of clinically related drug resistance.

## Data Availability

The original contributions presented in the study are included in the article/Supplementary Material, further inquiries can be directed to the corresponding authors.
